# MicroRNA‐26a alleviates tubulointerstitial fibrosis in diabetic kidney disease by targeting PAR4

**DOI:** 10.1111/jcmm.18099

**Published:** 2024-01-02

**Authors:** Gaoting Qu, Xingyue Li, Ran Jin, Dian Guan, Jialing Ji, Shanwen Li, Huimin Shi, Pingfan Tong, Weihua Gan, Aiqing Zhang

**Affiliations:** ^1^ Department of Pediatric Nephrology The Second Affiliated Hospital of Nanjing Medical University Nanjing P.R. China; ^2^ Department of Pediatric Surgery The First Affiliated Hospital of Nanjing Medical University Nanjing P.R. China; ^3^ Department of Pediatrics The Fourth Affiliated Hospital of Nanjing Medical University Nanjing P.R. China

**Keywords:** diabetic kidney disease, microRNA‐26a, protease‐activated receptor 4, tubulointerstitial fibrosis

## Abstract

Our previous study found that miR‐26a alleviates aldosterone‐induced tubulointerstitial fibrosis (TIF). However, the effect of miR‐26a on TIF in diabetic kidney disease (DKD) remains unclear. This study clarifies the role and possible mechanism of exogenous miR‐26a in controlling the progression of TIF in DKD models. Firstly, we showed that miR‐26a was markedly decreased in type 2 diabetic db/db mice and mouse tubular epithelial cells (mTECs) treated with high glucose (HG, 30 mM) using RT‐qPCR. We then used adeno‐associated virus carrying miR‐26a and adenovirus miR‐26a to enhance the expression of miR‐26a in vivo and in vitro. Overexpressing miR‐26a alleviated the TIF in db/db mice and the extracellular matrix (ECM) deposition in HG‐stimulated mTECs. These protective effects were caused by reducing expression of protease‐activated receptor 4 (PAR4), which involved in multiple pro‐fibrotic pathways. The rescue of PAR4 expression reversed the anti‐fibrosis activity of miR‐26a. We conclude that miR‐26a alleviates TIF in DKD models by directly targeting PAR4, which may provide a novel molecular strategy for DKD therapy.

## 1 INTRODUCTION

Diabetic kidney disease (DKD) is the main cause of chronic kidney disease (CKD) and end‐stage renal disease (ESRD) requiring dialysis or kidney transplantation worldwide.^1^, ^2^, ^3^ Tubulointerstitial fibrosis (TIF) is widely recognized as an essential pathway for the progression of DKD to ESRD and is accompanied by tubular injury and extracellular matrix (ECM) deposition.^4^, ^5^ Effective strategies for the treatment of TIF are urgently needed.

MicroRNAs (miRNAs) are highly conserved, endogenous non‐coding RNAs that have been proven to be involved in the pathogenesis of multiple kidney diseases.^6^, ^7^ Emerging evidence suggests a tight connection between renal fibrosis and certain miRNAs. Specifically, miR‐21was found increased in mice with renal fibrosis and knockdown of miR‐21 mitigates fibrosis in DKD.^8^ Another research illustrates that miR‐30 and miR‐146a serve as antifibrotic roles in DKD.^6^ These data suggest that targeting specific miRNAs could be a promising therapeutic strategy for renal fibrosis in DKD. Our previous study found that miRNA‐26a expression levels are diminished in both heart and kidney tissues of CKD mice, and exogenous application of miR‐26a depressed renal fibrosis in CKD mice by targeting connective tissue growth factor (CTGF).^9^, ^10^ Of note, we also revealed that exogenous miR‐26a can ameliorate ALD‐induced TIF.^11^ Despite this, the role miR‐26a on TIF of DKD remains unclear.

We used bioinformatics analysis to screen the target mRNAs of miR‐26a and found that miR‐26a has potential binding sites in the 3′‐UTR of protease‐activated receptor 4 (PAR4). As a subfamily of G‐protein‐coupled receptors, PAR4 has been reported to be involved in the regulation of various diseases,^12^, ^13^, ^14^, ^15^ but its contribution to kidney diseases remaining unknown. In the current study, we analysed the expression of miR‐26a in DKD models and further investigated that miR‐26a improves TIF in DKD via silencing PAR4. Our results will provide new insights into the pathogenesis and treatment of DKD.

## 2 MATERIALS AND METHODS

### 2.1 Animal and treatment

Eight‐week‐old male C57BLKS/J Lepr background db/db mice (40–43 g) were used as a type 2 diabetes model, and their heterozygous 8‐week‐old male db/m mice (19–21 g) served as controls. Mice were purchased from GemPharmatech LLC (Nanjing, China) at the same time and were fed adaptively at 23 ± 2°C, 55% ± 5% relative humidity, with 12‐h of light and 12‐h of darkness under specific pathogen‐free conditions. The db/db mice developed hyperglycemia from about 8 weeks of age, and the blood glucose was increased to more than 28.6 ± 13.2 mM at 20 weeks of age,^16^ accompanied by deterioration of renal function，renal hypertrophy, diffuse expansion of mesangial matrix and TIF.^17^ Therefore, db/db mice are a classic tool for studying human DKD. At 12 weeks of age, adeno‐associated virus miR‐26a (AAV‐miR‐26a, GenePharma, Shanghai, China) was used to overexpress miR‐26a, and adeno‐associated virus GFP (AAV‐GFP, GenePharma, Shanghai, China) was used as a negative control (NC) in mice. The db/m mice were randomly divided into the following groups (*n* = 6 per group): db/m group, db/m + miR‐26a group (a single injection by tail vein with le + 12 vg AAV‐miR‐26a at 12 weeks of age), db/m + NC group (a single injection by tail vein with le + 12 vg AAV‐GFP at 12 weeks of age). The db/db mice were divided into the following groups (*n* = 6 per group): db/db group, db/db + miR‐26a group (a single injection by tail vein with le + 12 vg AAV‐miR‐26a at 12 weeks of age), db/db + NC group (a single injection by tail vein with le + 12 vg AAV‐GFP at 12 weeks of age). All mice were anaesthetized with isoflurane and sacrificed at 20 weeks of age. Urine samples, serum samples and renal tissues were collected for further experiments. All animal experiments were approved by the Institutional Animal Care and Use Committee of Nanjing Medical University (No. 2101016).

### 2.2 Cell culture and transfection

Mouse tubular epithelial cells (mTECs) were gifted by Zhongda Hospital Southeast University. Human embryonic kidney (HEK) 293T cells were purchased from Procell Life Science &Technology Co., Ltd (Wuhan, China) for luciferase binding experiments. Both cell lines were cultured in Dulbecco's modified eagle medium (DMEM) containing 10% foetal bovine serum (FBS) at 37°C in 5% CO_2_. The mTECs in high glucose (HG) group were treated with 30 mM glucose for 24 h, 36 h or 48 h, while the cells in normal glucose (NG) group were treated with 5 mM glucose.

Adenovirus miR‐26a (Ad‐miR‐26a, GenePharma, Shanghai, China) was used to overexpress miR‐26a, and adenovirus miR‐NC (Ad‐miR‐NC, GenePharma, Shanghai, China) was used as a negative control in mTECs. The mTECs were divided into four groups: NG + NC (NG + Ad‐miR‐NC), NG + miR‐26a (NG + Ad‐miR‐26a), HG + NC (HG + Ad‐miR‐NC) and HG + miR‐26a (HG + Ad‐miR‐26a).

Protease‐activated receptor 4 (PAR4) plasmids were constructed by RiboBio (Guangzhou, China) to allow for the overexpression of PAR4, and the vectors were used as negative control in HG‐treated mTECs. HG + miR‐26a + vector group: HG‐treated mTECs transfected with Ad‐miR‐26a and vectors. HG + miR‐26a + PAR4 group: HG‐treated mTECs transfected with Ad‐miR‐26a and PAR4 plasmids.

### 2.3 Blood and urine determination

The levels of urinary protein and serum creatinine (Scr) were measured using a commercial kit (C035‐2‐1, Jiancheng, Nanjing, China) and a creatinine kit (C011‐2‐1, Jiancheng, Nanjing, China) following the manufacturer's instructions.^18^

### 2.4 Masson trichrome staining and immunohistochemistry

Paraffin‐embedded sections containing kidney tissues were was assessed using a Masson's Trichrome Staining Kit (G1340, Solarbio, China) following the manufacturer's instructions.^11^ Immunohistochemistry was performed using the biotin‐streptavidin‐peroxidase method (KTT‐9710, MXB Biotechologles, China) following the manufacturer's instructions.^11^ The primary antibodies was rabbit anti‐FN (1:150, ab2413, Abcam, UK). The sections were observed using an inverted microscope.

### 2.5 Immunofluorescence

Paraffin‐embedded sections were hydrated after dewaxed at 65°C for 2 h, then boiled in citrate buffer and cooled naturally to room temperature (RT). After blocking with 5% BSA at RT for 1 h, the primary antibody mouse anti α‐smooth muscle actin (anti‐α‐SMA, 1:200, AF1032, Affinity Biosciences, China) or rabbit anti‐COL I (1:100, ab34710, Abcam, UK) was added at 4°C overnight. Alexa Fluor® 647 conjugated secondary antibody (goat anti‐mouse IgG H&L, 1:400, ab150115, Abcam, UK) and Alexa Fluor® 488 conjugated secondary antibody (goat anti‐rabbit IgG H&L, 1:300, AFSA005, AiFang biological, China) were added at RT for 1 h. Then, the sections was stained with DAPI at RT for 10 min. Finally, the sections were observed using a fluorescence microscope.

### 2.6 Luciferase reporter assay

PAR4‐3′UTR wild‐type (WT) and PAR4‐3′UTR mutant (MUT) luciferase reporter plasmids were constructed and verified by GenePharma (Shanghai, China). The miR‐26a mimic was used to overexpress miR‐26a, and miR‐NC was used as a negative control. For the luciferase assay, PAR4 3′‐UTR‐WT or PAR4 3′‐UTR‐MUT plasmids were co‐transfected with miR‐26a mimic or miR‐NC into 293T cells or mTECs using Lipofectamine 2000 (Thermo Fisher Scientific, USA). After incubation for 48 h, the luciferase activity was detected using a luciferase assay kit (GenePharma, Shanghai, China).

### 2.7 Western blot

Western blot was conducted and analysed as previously described.^9^ Proteins (50 μg) were separated by 8%, 10% and 12% SDS‐PAGE, respectively, according to molecular weight. The primary antibodies used were as following: GAPDH (1:1000, T0004, Affinity Biosciences, China), kidney injury molecule 1 (KIM‐1, 1:1000, AF1817, R&D Systems, USA), α‐SMA (1:1000, AF1032, Affinity Biosciences, China), FN (1:1000, ab2413, Abcam, UK), COL I (1:1000, ab138492, Abcam, UK), PAR4 (1:1000, AF5371, Affinity Biosciences, China). The secondary antibodies used were as following: goat anti‐mouse (1:2000, L3032, Signalway Antibody Co., China) and goat anti‐Rabbit (1:2000, L3012, Signalway Antibody Co., China).

### 2.8 Real‐time quantitative PCR (RT‐qPCR)

Total RNAs (including mRNA and microRNA) was purified from kidney and mTECs using TRIzol® reagent (Life Technologies; Agilent, Inc., USA). RT‐qPCR was performed and analysed as described previously.^19^ The primers sequences are shown in Table 1. The thermocycling conditions were presented in Tables 2 and 3.

**TABLE 1:** Sequences of the primer sequences used for reverse transcription‐quantitative PCR.

Genes	Forward primer (5′‐3′)	Reverse primer (5′‐3′)
miR‐26a	ACACTCCAGCTGGGTTCAAGTAATCCAGGA	TGGTGTCGTGGAGTCG
U6	CTCGCTTCGGCAGCACATATACT	ACGCTTCACGAATTTGCGTGTC
KIM‐1	CTATGTTGGCATCTGCATCG	AAGGCAACCACGCTTAGAGA
FN	GCAAGAAGGACAACCGAGGAAA	GGACATCAGTGAAGGAGCCAGA
COL I	GTCAGACCTGTGTGTTCCCTACTCA	TCTCTCCAAACCAGACGTGTTC
α‐SMA	CAGCAAACAGGAATACGACGAA	AACCACGAGTAACAAATCAAAGC
PAP4	GATGAGCCTGAGTATGAGGATGG	CAAGACACAATTCGGCCTGG
β‐actin	CATCCGTAAAGACCTCTATGCCAAC	ATGGAGCCACCGATCCACA

**TABLE 2:** Thermocycling conditions used during reverse transcription‐quantitative PCR for microRNA expression level evaluation.

Stage	Step	Repetition	Temperature (°C)	Duration
Stage 1	Initial denaturation	1	95	10 min
Stage 2	Amplification cycles	40	95 60 72	10 sec 20 sec 10 sec
Stage 3	Dissociation curve	1	95	15 sec

**TABLE 3:** Thermocycling conditions used during reverse transcription‐quantitative PCR for mRNA expression level evaluation.

Stage	Step	Repetition	Temperature (°C)	Duration
Stage 1	Initial denaturation	1	95	30 sec
Stage 2	Amplification cycles	40	95 60	10 sec 30 sec
Stage 3	Dissociation curve	1	95 60 95	15 sec 60 sec 15 sec

### 2.9 Bioinformatical analysis

TargetScan (http://www.targetscan.org/) and miRDB (http://mirdb.org/) were employed to predict miR‐26a‐target genes.

### 2.10 Statistical analysis

All data analyses were assessed with the GraphPad Prism 9.3.1 software (GraphPad Software, Inc., California, USA). The data are presented as mean ± SD of at least three independent experiments. The data are analysed using unpaired Student's *t*‐test (two groups) and one‐way ANOVA (three or more groups), followed by a Tukey's post hoc test. *p*‐value <0.05 were considered to indicate a significant difference.

## 3 RESULTS

### 3.1 Expression of miR‐26a decreased and TIF aggravated in diabetic mice

**FIGURE 1:**
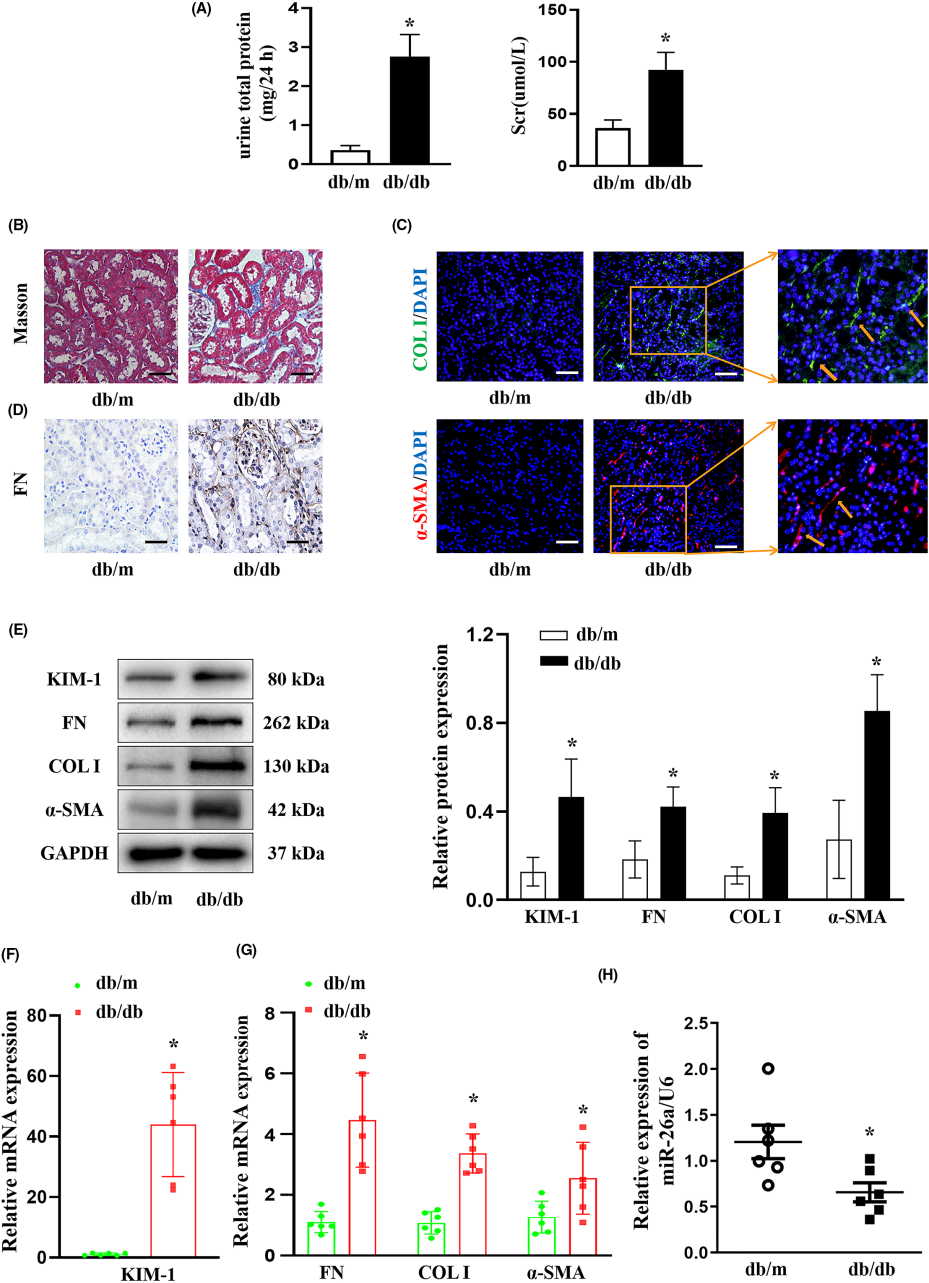
mir‐26a expression was reduced and TIF was aggravated in DKD mice. (A) Scr and 24‐h urine total protein in the mice at 20 weeks of age. *n* = 6 per group. (B) Representative images of Masson staining in the kidney tissue of mice. Scale bar, 50 μm. (C) Representative images of COL I (green) and α‐SMA (red) expression assessed by immunofluorescence analyses in the kidney tissue of mice. DAPI (blue) was used to stain the nuclei. Scale bar, 50 μm. (D) Representative images of FN expression assessed by immunohistochemistry analyses in the kidney tissue of mice. (E) Western blot analyses of KIM‐1, FN, COL I and α‐SMA protein expressions in the kidney tissue of mice. (F) RT‐qPCR analysis of KIM‐1 in the kidney tissue of mice. (G) RT‐qPCR analyses of FN, COL I and α‐SMA in the kidney tissue of mice. (H) RT‐qPCR analysis of miR‐26a in the kidney tissue of mice. All data are expressed as mean ± SD; *n* = 6 in each group. *p < 0.05 versus db/m group. miR, microRNA; TIF, tubulointerstitial fibrosis; DKD, diabetic kidney disease; Scr, serum creatinine, COL I, collagen I; α‐SMA, α‐smooth muscle Actin; KIM‐1, kidney injury molecule 1; FN, fibronectin; RT‐qPCR, reverse transcription‐quantitative PCR.

To explore the role of miR‐26a in TIF of DKD, a type 2 diabetic model with db/db mice was first established to simulate human diabetic kidney disease. As shown in Figure 1A, 24 h‐UTP and serum creatinine (Scr) levels were markedly increased in db/db mice at 20 weeks of age. Histologically, masson staining showed an increase in tubulointerstitial collagen expression in db/db group (Figure 1B). Additionally, the expression of the fibrotic markers COL I, α‐SMA and FN were upregulated in the kidneys of db/db group on immunofluorescence (Figure 1C) and immunohistochemistry (Figure 1D). Consistently, Western blot (Figure 1E) and RT‐qPCR (Figure 1F,G) assays depicted that the tubular injury marker KIM‐1 and fibrotic markers (COL I, α‐SMA and FN) were higher in the kidneys of db/db group. Furthermore, we observed that the miR‐26a level in the kidney tissue of db/db mice considerably decreased contrast with db/m group (Figure 1H). These results suggest that the reduced miR‐26a expression may contribute to TIF in DKD.

### 3.2 Expression of miR‐26a decreased and ECM deposition increased in mTECs under HG condition

**FIGURE 2:**
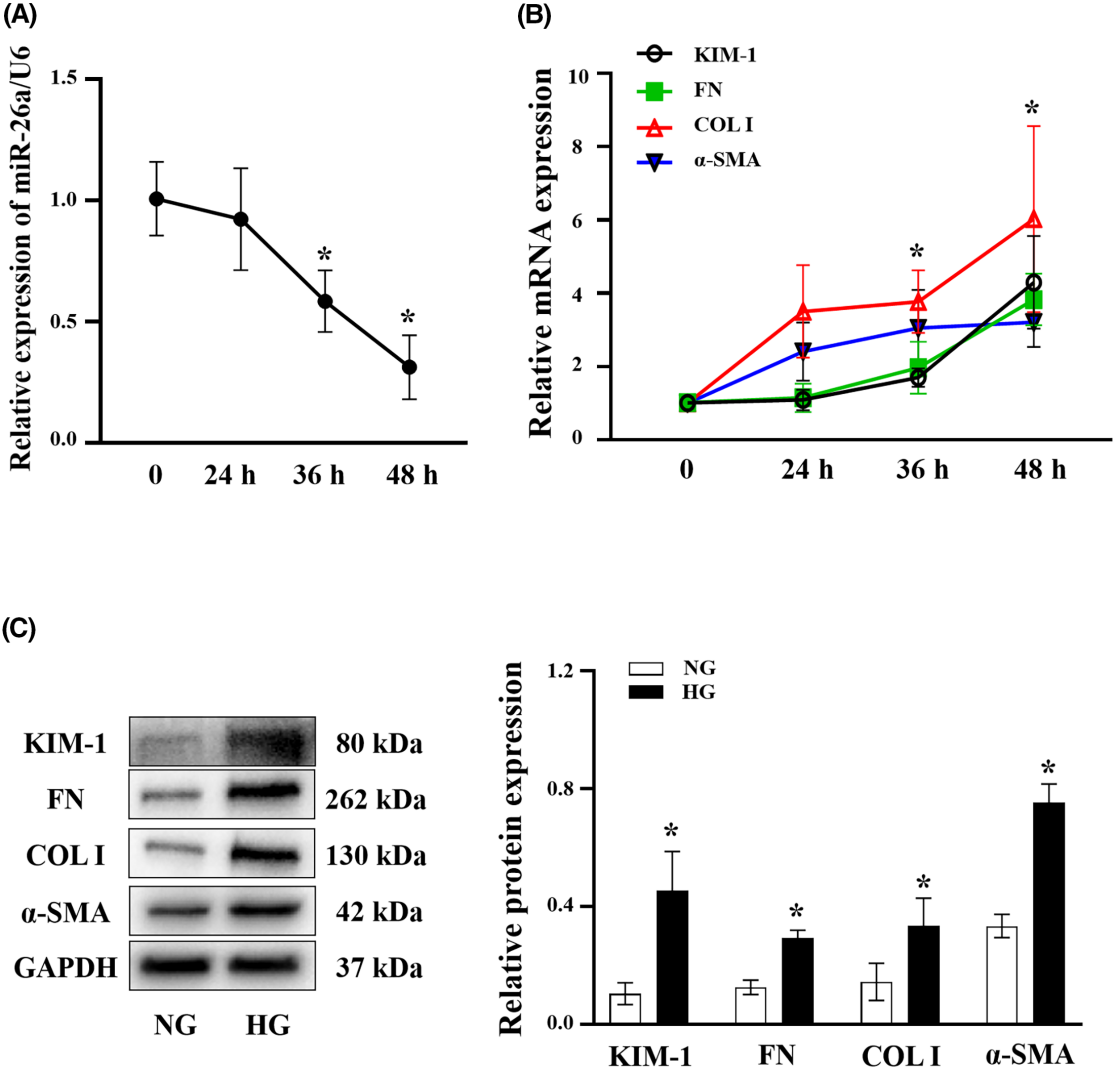
Downregulation of miR‐26a and upregulation of ECM proteins in HG‐treated mTECs. (A) RT‐qPCR analyses of miR‐26a in mTECs treated with HG (30 mM) for 24 h, 36 h and 48 h. (B) RT‐qPCR analyses of KIM‐1, FN, COL I and a‐SMA in mTECs treated with HG for 24 h, 36 h and 48 h. (C) Western blot analyses of KIM‐1, FN, COL I and a‐SMA protein expressions in mTECs treated with HG for 48 h. All data are expressed as mean ± SD; *n* = 3 in each group. *p < 0.05 versus NG group. miR, microRNA; ECM, extracellular matrix; HG, high glucose; NG, normal glucose; mTECs, mouse tubular epithelial cells; COL I, collagen I; α‐SMA, α‐smooth muscle Actin; KIM‐1, kidney injury molecule 1; FN, fibronectin; RT‐qPCR, reverse transcription‐quantitative PCR.

Given that tubular epithelial cells (TECs) play a key role in the process of TIF in DKD,^5^ we next established an in vitro model by stimulating mTECs with high glucose (HG, 30 mM glucose). RT‐qPCR data depicted a descended miR‐26a expression and ascended expressions of tubular injury marker KIM‐1 and fibrosis‐related proteins (FN, COL I and a‐ SMA) in mTECs treated with HG for 24 h, 36 h and 48 h (Figure 2A,B). The above‐mentioned expression changed more significantly when the intervention time was 48 h. Therefore, 48 h of HG treatment was conducted thereafter. On Western blot, the expression of KIM‐1, FN, COL I and a‐SMA were increased in the HG group compared with the NG group (Figure 2C). These data suggest that HG‐induced ECM deposition in mTECs may be related to downregulated miR‐26a expression levels.

### 3.3 Overexpression of miR‐26a alleviated TIF in diabetic mice

**FIGURE 3:**
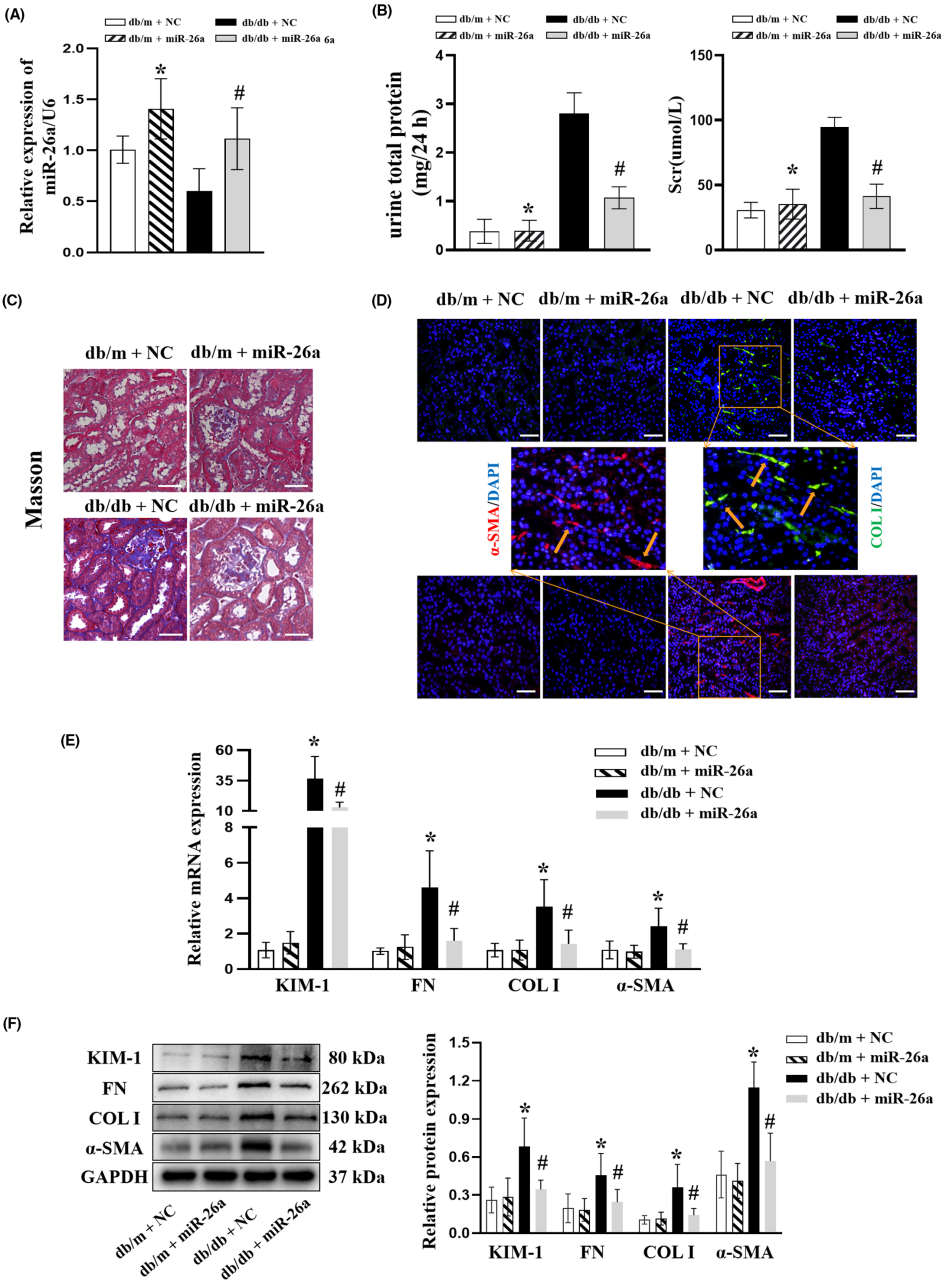
Mir‐26a overexpression alleviates TIF in DKD model. db/m and db/db mice were injected with AAV‐miR‐26a or NC at 12 weeks of age, and were sacrificed at 20 weeks of age. (A) RT‐qPCR analysis of miR‐26a in the kidney tissue of mice. (B) Scr and 24‐h urine total protein in the mice at 20 weeks of age. (C) Representative images of Masson staining in mice. Scale bar, 50 μm. (D) Representative images of COL I (green) and α‐SMA (red) expression assessed by immunofluorescence analyses in the kidney tissue of mice. DAPI (blue) was used to stain the nuclei. Scale bar, 50 μm. (E) RT‐qPCR analyses of KIM‐1, FN, COL I and a‐SMA in the kidney tissue of mice. (F) Western blot analyses of KIM‐1, FN, COL I and a‐SMA protein expressions in the kidney tissue of mice. All data are expressed as mean ± SD; *n* = 6 in each group. *p < 0.05 versus db/m + NC group. ^#^ *p* < 0.05 versus db/db + NC group. miR, microRNA; TIF, tubulointerstitial fibrosis; DKD, diabetic kidney disease; COL I, collagen I; α‐SMA, α‐smooth muscle Actin; KIM‐1, kidney injury molecule 1; FN, fibronectin; RT‐qPCR, reverse transcription‐quantitative PCR; NC, negative control.

To further investigate the effect of ectogenous miR‐26a on TIF in DKD, db/db mice were injected with AAV‐miR‐26a (le + 12 vg) at 12 weeks of age and were sacrificed at 20 weeks of age. The exogenously added AAV‐miR‐26a replenished miR‐26a in the kidney of the db/db mice (Figure 3A). Elevated Scr and 24h‐UTP levels in db/db mice were significantly ameliorated by AAV‐miR‐26a treatment (Figure 3B). Further, masson staining showed a reduction in tubulointerstitial collagen expression in db/db + miR‐26a group (Figure 3C). In addition, immunofluorescence revealed that COL I and a‐SMA was remarkably attenuated by miR‐26a overexpression in dbdb mice (Figure 3D). Moreover, the expressions of KIM‐1, FN, COL I and α‐SMA were markedly reversed by AAV‐miR‐26a treatment in db/db mice as shown by RT‐qPCR (Figure 3E), consistent with the Western blot results (Figure 3F). The above results demonstrated that exogenous miR‐26a inhibited TIF in DKD.

### 3.4 Overexpression of miR‐26a reduced HG‐induced ECM deposition in mTECs

**FIGURE 4:**
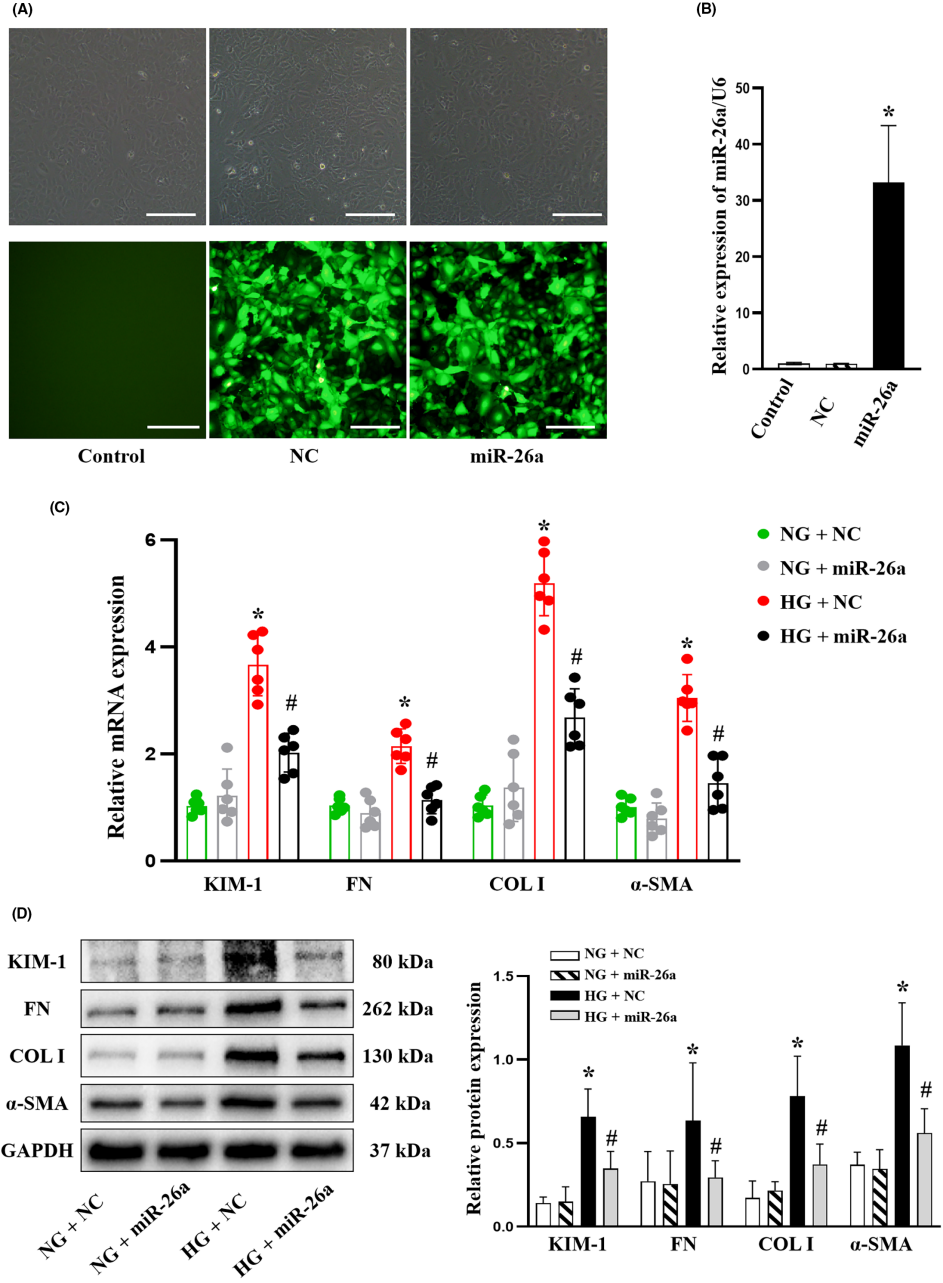
Mir‐26a overexpression alleviates HG‐induced ECM deposition in mTECs. (A) The expression of miR‐26a in mTECs transfected with miR‐26a (Ad‐miR‐26a) or NC (Ad‐miR‐NC) is determined using immunofluorescence. Scale bar, 50 μm. (B) RT‐qPCR analysis of miR‐26a in mTECs transfected with miR‐26a or NC. *p < 0.05 versus Control. (C) RT‐qPCR analyses of KIM‐1, FN, COL I and a‐SMA in mTECs transfected with miR‐26a or NC. *p < 0.05 versus NG + NC group. ^#^ *p* < 0.05 versus HG + NC group. (D) Western blot analyses of KIM‐1, FN, COL I and a‐SMA protein expressions in mTECs transfected with miR‐26a or NC. All data are presented as mean ± SD; *n* = 3 in each group. *p < 0.05 versus NG + NC group. ^#^ *p* < 0.05 versus HG + NC group. miR, microRNA; HG, high glucose; NG, normal glucose; ECM, extracellular matrix; mTECs, mouse tubular epithelial cells; COL I, collagen I; α‐SMA, α‐smooth muscle Actin; KIM‐1, kidney injury molecule 1; FN, fibronectin; RT‐qPCR, reverse transcription‐quantitative PCR; NC, negative control.

To characterize the biological functions of exogenous miR‐26a in ECM accumulation, mTECs were treated with Ad‐miR‐26a to enhance the expression of miR‐26a (Figure 4A,B). Furthermore, as shown by the results of RT‐qPCR (Figure 4C) and Western blot (Figure 4D), Ad‐miR‐26a could dramatically inhibit the expression of KIM‐1 and fibrosis‐related proteins (FN, COL I and α‐SMA). In conclusion, exogenous miR‐26a prevented ECM accumulation triggered by high glucose in mTECs.

### 3.5 Elevated PAR4 in mice and mTECs under diabetic condition

**FIGURE 5:**
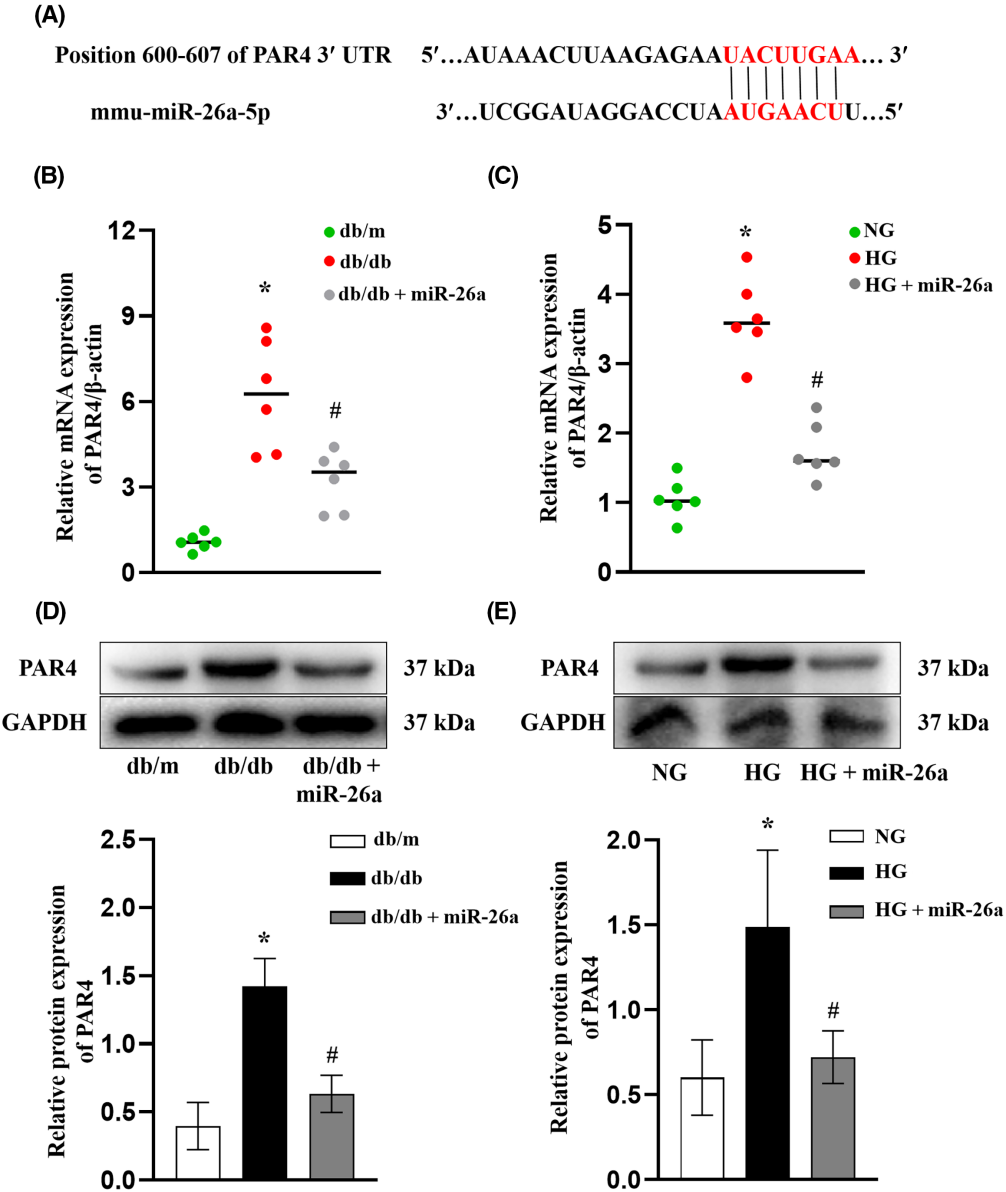
PAR4 Levels incresed in mice and mTECs under diabetic condition. (A) miR‐26a target site in the 3′UTR of PAR4. (B) RT‐qPCR analysis of PAR4 mRNA expression in the kidney tissue of mice. *p < 0.05 versus db/m group. ^#^ *p* < 0.05 versus db/db group. (C) RT‐qPCR analysis of PAR4 in mTECs. *p < 0.05 versus NG group. ^#^ *p* < 0.05 versus HG group. (D) Western blot analysis of PAR4 protein expression in the kidney tissue of mice. *p < 0.05 versus db/m group. ^#^ *p* < 0.05 versus db/db group. (E) Western blot analysis of PAR4 protein expression in mTECs. *p < 0.05 versus NG group. ^#^ *p* < 0.05 versus HG group. All data are presented as mean ± SD; *n* = 3 in each group. miR, microRNA; HG, high glucose; NG, normal glucose; mTECs, mouse tubular epithelial cells; PAR4; protease‐activated receptor 4; KIM‐1, kidney injury molecule 1; FN, fibronectin; COL I, collagen I; α‐SMA, α‐smooth muscle Actin; RT‐qPCR, reverse transcription‐quantitative PCR; NC, negative control.

With the attempt to explore the molecular mechanism of the antifibrotic activity of miR‐26a, we used bioinformatics databases (TargetScan and miRDB) to predict the target genes of miR‐26a and found that there existed the presence of binding sites between miR‐26a and the 3′UTR of PAR4 (Figure 5A). Considering that PAR4 was reported to be involve in the fibrosis process in various organs,^20^ we hypothesized that PAR4 might be a potential target of miR‐26a. As expected, RT‐qPCR (Figure 5B) and Western blot (Figure 5D) results revealed higher level of PAR4 in db/db group compared with db/m group. When miR‐26a was overexpressed in db/db mice, PAR4 level was downregulated (Figure 5B,D). Consistently, PAR4 levels were increased in HG‐induced mTECs (Figure 5C,E) and were decreased following the ectopic expression of miR‐26a by Ad‐miR‐26a (Figure 5C,E). These data indicated that the increased PAR4 expression may be responsible for in DKD and be negatively regulated by miR‐26a.

### 3.6 Ectogenous miR‐26a alleviates HG‐induced ECM deposition in mTECs by targeting PAR4

**FIGURE 6:**
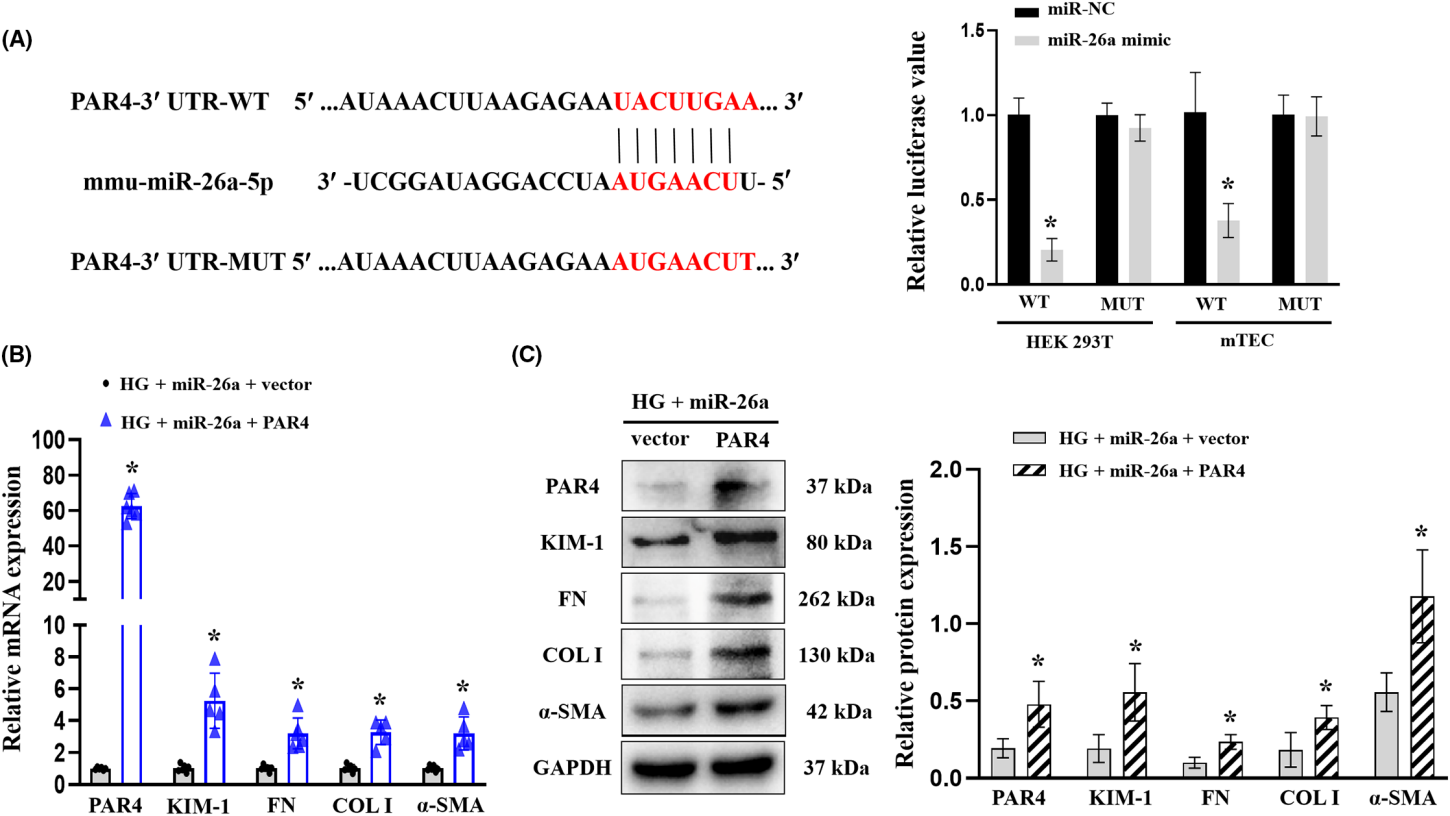
miR‐26a directly targets PAR4 and alleviates ECM deposition in mTECs. (A) The luciferase activity of PAR4‐3′‐UTR‐WT (WT) and PAR4‐3′‐UTR‐MUT (MUT) in HEK‐293 T cells and mTECs co‐transfected with miR‐26a mimics or miR‐NC. *p < 0.05 versus miR‐NC. (B) RT‐qPCR analyses of PAR4, KIM‐1, FN, COL I and a‐SMA in HG‐treated mTECs transfected with miR‐26a and PAR4‐overexpression plasmid. *p < 0.05 versus HG + miR‐26a + vector group. (C) Western blot analyses of PAR4, KIM‐1, FN, COL I and a‐SMA protein expression levels in mTECs HG‐treated mTECs transfected with miR‐26a and PAR4‐overexpression plasmid. *p < 0.05 versus HG + miR‐26a + vector group. All data are presented as mean ± SD; *n* = 3 in each group. miR, microRNA; HG, high glucose; mTECs, mouse tubular epithelial cells; PAR4; protease‐activated receptor 4; KIM‐1, kidney injury molecule 1; FN, fibronectin; COL I, collagen I; α‐SMA, α‐smooth muscle actin; RT‐qPCR, reverse transcription‐quantitative PCR; NC, negative control.

To certificate whether miR‐26a could directly target PAR4 mRNA, luciferase reporters containing either wild‐type (WT) or mutated‐type (MUT) miR‐26a binding sites at the 3′UTR of PAR4 were constructed and transfected into mTECs (Figure 6A). Inspiringly, luciferase activity was markedly reduced in miR‐26a‐mimic and PAR4‐3′‐UTR‐WT transfected cells compared with that in miR‐NC and PAR4‐3′‐UTR‐WT transfected cells, and the luciferase activity was not suppressed significantly in PAR4‐3′‐UTR‐MUT transfected cells (Figure 6A). Moreover, RT‐qPCR (Figure 6B) and Western blot (Figure 6C) analysis showed that overexpression of PAR4 significantly counteracted the protective effect of miR‐26a on HG‐induced ECM deposition in mTECs, as evidenced by the increased levels of KIM‐1, FN, COL I and α‐SMA. The obtained data indicate that miR‐26a may inhibit the progression of TIF in DKD by directly downregulating PAR4.

## 4 DISCUSSION

**FIGURE 7:**
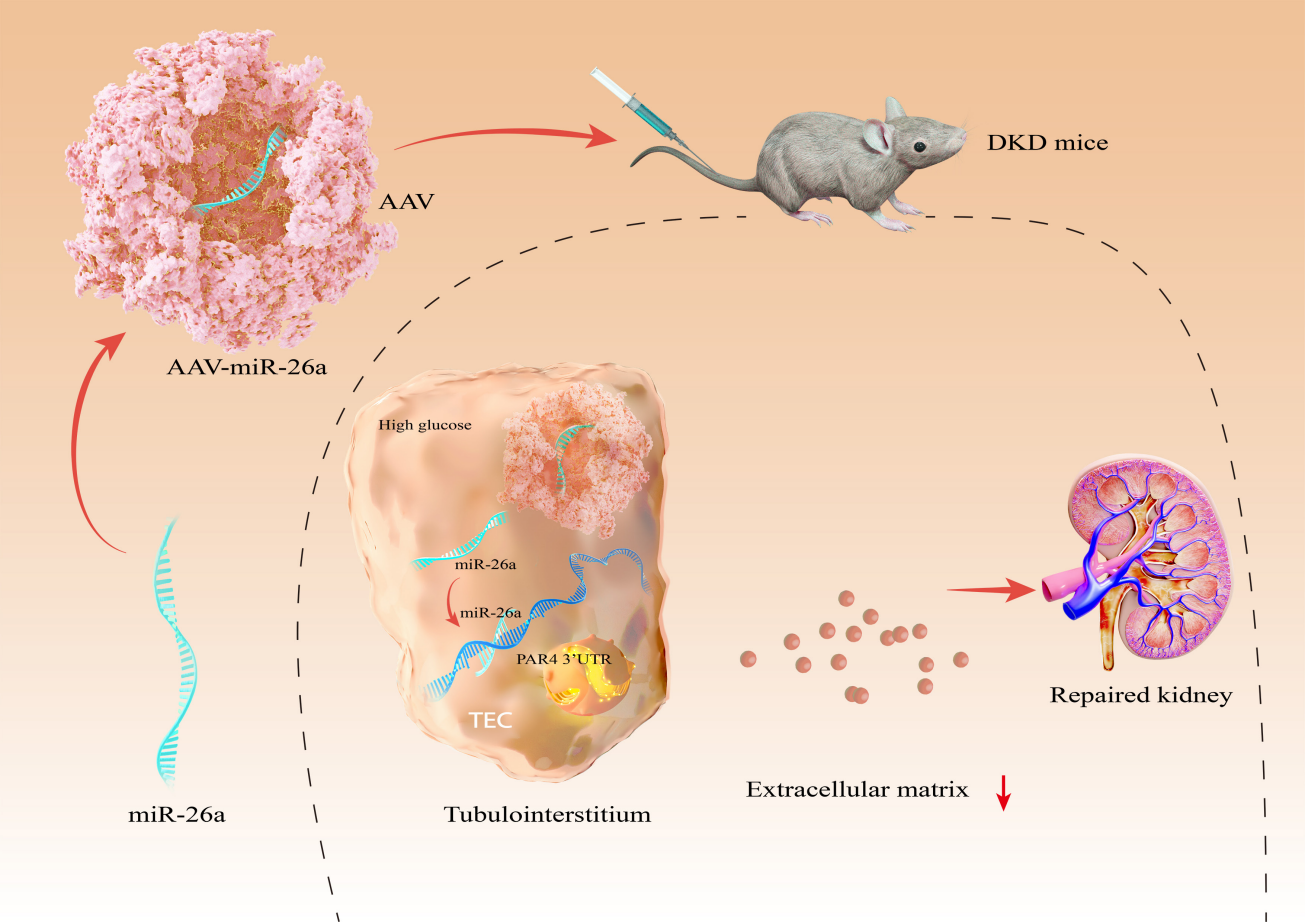
Schematic illustration of ectogenous miR‐26a for the treatment of DKD. Exogenous miR‐26a through the tail vein in mice ameliorate tubular injury and TIF by a mechanism that directly targets PAR4.

DKD is a serious complication of diabetes that results in kidney function loss. TIF correlates the best with DKD progression.^21^ The involvement of miRNAs in the progression of DKD has been proposed in previous studies.^22^, ^23^ The present study demonstrates that ectogenic miR‐26a effectively alleviated renal TIF in diabetic mice, and overexpression of miR‐26a significantly ameliorated ECM deposition in mTECs via suppressing PAR4 expression (Figure 7). These findings may provide a promising approach for the treatment of TIF in DKD using exogenous miR‐26a.

miR‐26a is a highly conserved, small RNA that acts as a regulator in multiple cardiovascular and tumour‐related diseases, including myocardial infarction, cardiac hypertrophy, colorectal cancer and osteosarcoma.^24^, ^25^, ^26^, ^27^, ^28^ A previous study demonstrated that miR‐26a‐5p protects cardiomyocytes from damage caused by hypoxia/reoxygenation through the negative regulation of WNT5A via the Wnt/β‐catenin signalling pathway.^24^ It was reported that miR‐26a‐5p directly targets ADAM17 to inhibit cardiac hypertrophy and dysfunction in mice with cardiac hypertrophy.^25^ miR‐26a enhances the tumorigenesis of colorectal cancer by decreasing the acetylation of RREB1 to activate the AKT signalling pathway.^26^ However, studies regarding the function of miR‐26a in kidney disease are limited. Chen et al. discovered that miR‐26a‐5p play a protective role in septic acute kidney injury.^29^ In addition, our previous research discovered that miR‐26a improved TGF‐β‐triggered ECM accumulation by silencing CTGF and that miR‐26a level was diminished in the glomeruli of diabetic mice.^10^ In this study, miR‐26a expression showed a remarkable attenuation in the renal tissue of db/db mice and HG‐treated mTECs. In addition, the overexpression of miR‐26a alleviated renal tubular injury and renal dysfunction in db/db mice, highly suggesting a role of miR‐26a in mediating the tubular function in DKD.

Growing evidence suggests that TIF is critic in the pathological progress of DKD. Recently, the emerging effect of miRNAs in the development of TIF has been highlighted. Specifically, miR‐30e reduces the progression of epithelial‐mesenchymal transdifferentiation in proximal tubular cell during TIF through blocking Snail, Slug and Zeb2 expression and rescuing E‐cadherin expression.^30^ miR‐192 function as a TIF repressor in DKD by targeting Egr1 to promote TGF‐β1 and FN degradation.^31^ miR‐22 is upregulated in the kidney tissues of rats with DKD and exerts autophagy‐inhibiting and TIF‐promoting effects by targeting phosphatase and tensin homologues.^32^ However, no biological effects of miR‐26a on TIF in DKD have been reported previously. In this study, we observed that miR‐26a overexpression protected diabetic mice from the development of TIF, with a remarkable drop in the expression of FN, COL I and α‐SMA in vivo and in vitro. These results revealed that increased miR‐26a levels may play a crucial role in ameliorating TIF in DKD.

Another interesting finding of our study was that miR‐26a targets PAR4. PAR4 is expressed in several types of cells and is an important mediator that facilitates direct communication between proteases and intracellular signalling mechanisms.^33^, ^34^ Previous studies have demonstrated that PAR4 can activate a variety of pro‐fibrotic signalling pathways, including MAPK, PIK3‐Akt, STAT and NF‐κB signalling pathways.^35^, ^36^ PAR4 is also involved in the pathophysiology of organ fibrosis, though few studies regarding this role of PAR4 have been reported. Joshi et al. found that PAR4 deficiency exacerbated alpha‐naphthylisothiocyanate‐induced liver fibrosis.^37^ On the other hand, studies by François et al. have shown that appropriate doses of PAR4 antagonists decrease progressive pulmonary fibrosis.^38^ However, whether PAR4 mediate the progression of renal fibrosis remained to be explored. Here, we showed that miR‐26a overexpression dramatically restrained PAR4 expression and then revealed that miR‐26a downregulated PAR4 by directly binding to the 3′ UTR of PAR4 mRNA. Furthermore, we found that PAR4 overexpression reversed the protective effect of exogenous miR‐26a on ECM deposition in HG condition. Collectively, the above results demonstrated that upregulating miR‐26a may inhibit the progression of TIF in DKD by targeting PAR4.

Nevertheless, there are still some limitations in this study. It remains to be established whether the similar results could be also observed in other cell lines or renal biopsies from patients with DKD. Additionally, further investigation is required to illustrate the downstream molecular mechanism of miR‐26a alleviating TIF in DKD.

In conclusion, the findings of our study is proposed for the first time that miR‐26a alleviates TIF progression in DKD by targeting PAR4. The current findings may not only provided new insights into the molecular mechanisms of TIF progression in DKD, but also opened new avenues for the treatment of renal fibrosis.

## AUTHOR CONTRIBUTIONS

**Gaoting Qu:** Data curation (equal); investigation (equal); methodology (equal); validation (equal); writing – original draft (equal). **Xingyue Li:** Data curation (equal); methodology (equal). **Ran Jin:** Software (equal). **Dian Guan:** Methodology (equal); software (equal). **Jialing Ji:** Data curation (equal). **Shanwen Li:** Writing – review and editing (equal). **Huimin Shi:** Writing – review and editing (equal). **Pingfan Tong:** Formal analysis (equal). **Weihua Gan:** Conceptualization (equal); writing – review and editing (equal). **Aiqing Zhang:** Conceptualization (equal); project administration (equal); writing – review and editing (equal).

## FUNDING INFORMATION

This work was fully supported by National Natural Science Foundation of China (81970664), Natural Science Foundation of Jiangsu Province (BK20191082 and BK20211385), 789 Outstanding Talent Program of SAHNMU (789ZYRC202080119 and 789ZYRC202090251), Science and Technology Development Foundation of Nanjing Medical University (NMUB2020052).

## CONFLICT OF INTEREST STATEMENT

The authors declare that there are no conflicts of interest.

## INSTITUTIONAL REVIEW BOARD STATEMENT

The experimental procedures in the present study were approved by Nanjing Medical University (IACUC‐2101016, Nanjing, China).

## Data Availability

The datasets used and/or analysed during the current study are available from the corresponding author on reasonable request.
